# Metformin-Mediated Improvement in Solubility, Stability, and Permeability of Nonsteroidal Anti-Inflammatory Drugs

**DOI:** 10.3390/pharmaceutics16030382

**Published:** 2024-03-11

**Authors:** Qi An, Cheng Xing, Zhipeng Wang, Shuang Li, Wenwen Wang, Shiying Yang, Linglei Kong, Dezhi Yang, Li Zhang, Guanhua Du, Yang Lu

**Affiliations:** 1 Beijing City Key Laboratory of Polymorphic Drugs, Center of Pharmaceutical Polymorphs, Institute of Materia Medica, Chinese Academy of Medical Sciences and Peking Union Medical College, Beijing 100050, China; a17861121095@163.com (Q.A.); xingc@imm.ac.cn (C.X.); zp091215@hotmail.com (Z.W.); lishuang990515@163.com (S.L.); wang987214342@163.com (W.W.); ysy@imm.ac.cn (S.Y.); luy@imm.ac.cn (Y.L.); 2 Beijing Key Laboratory of Drug Targets Identification and Drug Screening, National Center for Pharmaceutical Screening, Institute of Materia Medica, Chinese Academy of Medical Sciences and Peking Union Medical College, Beijing 100050, China; konglinglei@imm.ac.cn (L.K.); dugh@imm.ac.cn (G.D.)

**Keywords:** NSAIDs, metformin, drug–drug salt, theoretical calculation

## Abstract

Nonsteroidal anti-inflammatory drugs (NSAIDs) are class II biopharmaceutics classification system drugs. The poor aqueous solubility of NSAIDs can lead to limited bioavailability after oral administration. Metformin (MET), a small-molecule compound, can be used in crystal engineering to modulate the physicochemical properties of drugs and to improve the bioavailability of orally administered drugs, according to the literature research and preliminary studies. We synthesized two drug–drug molecular salts (ketoprofen–metformin and phenylbutazone–metformin) with NSAIDs and thoroughly characterized them using SCXRD, PXRD, DSC, and IR analysis to improve the poor solubility of NSAIDs. In vitro evaluation studies revealed that the thermal stability and solubility of NSAIDs-MET were substantially enhanced compared with those of NSAIDs alone. Unexpectedly, an additional increase in permeability was observed. Since the structure determines the properties, the structure was analyzed using theoretical calculations to reveal the intermolecular interactions and to explain the reason for the change in properties. The salt formation of NSAIDs with MET could substantially increase the bio-absorption rate of NSAIDs, according to the in vivo pharmacokinetic findings, which provides an experimental basis for developing new antipyretic and analgesic drugs with rapid onset of action.

## 1. Introduction

Antibiotics, antipyretics, anti-inflammatory and anticancer drugs, hormones, and endocrine function regulators are commonly administered drugs in clinical practice. Although some are used as first-line drugs in clinical treatment with improved pharmacological effects and low adverse reactions, they have defects in their physicochemical properties, such as low solubility, poor permeability, strong moisture attraction, poor mechanical properties, and thermal stability [1]. According to statistics, 40% of existing pharmaceutical products and approximately 90% of new chemicals have limited water solubility, resulting in low oral bioavailability and restricted routes of administration [2]. Thermal stability is a critical determinant in the process of drug development. Determining the temperature range in which a drug maintains its structure integrity and therapeutic effect is essential for optimizing storage conditions, facilitating technological advancements, and acquiring the appropriate formulation technology [3]. Additionally, gastrointestinal irritation is a common adverse effect of some medications; the topical or transdermal route of administration can help reduce this and shows improved therapeutic effects [4]. However, the stratum corneum still limits the penetration of these substances, resulting in limited bioavailability [5]. Self-emulsifying drug delivery systems, pH adjustment, particle size reduction, super-critical fluid processing, inclusion complexes, micelle solubilization, solid dispersions, nanosuspensions, cocrystals, salts, and nanocrystals are common formulation strategies to address these challenges [6,7,8].

In 2020, anti-inflammatory drugs were among the three most researched drug classes, accounting for 19% of all published papers, according to the Web of Science article database. Increasing numbers of scientific articles have demonstrated the significance of NSAIDs over the past decade [9]. Although NSAIDs differ considerably in their chemical class, they all inhibit prostaglandin production. This is accomplished through the inhibition of cyclooxygenase activity [10]. This group of drugs comprises antipyretic, anti-inflammatory, antiplatelet, antitumor, and analgesic drugs [11,12]. Although many drugs with a single therapeutic effect have been developed, such as antipyretic, analgesic, and anti-inflammatory drugs, NSAIDs are preferred and commonly administered due to their multiple therapeutic actions [13]. However, despite their high intestinal permeability, these class II biopharmaceutics classification system (BCS) drugs exhibit limited absorption in the stomach and intestines due to its poor water solubility. Thermodynamic studies reported that some NSAIDs also have poor thermal stability (e.g., ketoprofen and ibuprofen) [14].

Basic small molecules, including piperazine, betaine, ligustrazine, and metformin (MET), effectively modulate the physicochemical properties of acidic drugs. Of these small molecules, MET has undergone the most extensive research. It is an oral first-line drug for treating type 2 diabetes that prevents the liver from producing glucose by increasing insulin sensitivity in peripheral tissues and reducing hepatic gluconeogenesis [15]. Furthermore, MET has no hypoglycemic effect on normal people [16]. Metformin–hydrochloride (MET-HCl), chemically defined as a salt to enhance solubility and stability, is the most commonly prescribed formulation [17]. Clinical trial data have demonstrated that MET is safe for most individuals and unsafe for patients with severe hepatic, cardiac, or renal insufficiency [18].

Pharmaceutical cocrystals (or salts) are multiple-component systems with an active pharmaceutical ingredient (API) typically complexed in a stoichiometric ratio to cocrystal conformer (CCF) through non-covalent interactions like hydrogen bonding, Π-stacking, and dispersion forces. Increasing interest in the study of drug cocrystals and salts has been observed recently [19]. The big difference between a cocrystal and a salt is whether proton transfer occurs between the API and the CCF. Those with intermolecular proton transfer are called salts, while those without intermolecular proton transfer are cocrystals [20]. Salting can significantly improve solubility, stability, thermal properties, and other physicochemical properties, and it potentially improves in vivo bioavailability [21,22]. This approach is popular in the pharmaceutical industry as it improves the physicochemical properties of the API without changing its chemical structure compared to the complete drug development route.

Therefore, we used crystal engineering to synthesize drug–drug multi-component solids. Since MET is a strongly basic drug (pK_a_ = 12.4), the pK_a_ difference between the carboxyl groups with NSAIDs is >3. Thus, it is easy to form drug–drug salts [23,24,25]. We hope to use the excellent solubility of MET to improve the solubility and bio-absorption of NSAIDs. Diclofenac, niflumic acid, diflunisal, mefenamic acid, tolfenamic acid, and flurbiprofen are NSAIDs reported as salts with metformin [17,26]. These salts increased the solubility of NSAIDs; however, none were subjected to systematic pharmacokinetic experiments to evaluate their biological activity. Therefore, we prepared ketoprofen–metformin (KET-MET) and phenylbutazone–metformin (PBU-MET) using the liquid-assisted grinding method. We characterized the structure of NSAIDs-MET using the SCXRD, PXRD, FTIR, and DSC methods. Additionally, solubility and permeability evaluations were performed to investigate the improvement in the solubility and permeability of NSAIDs-MET. In vitro evaluation experiments showed that MET improved the thermal stability, solubility, and permeability of NSAIDs in drug molecular salts. The mechanism for improving these physicochemical properties was explained using theoretical calculations. The excellent improved physicochemical properties of NSAIDs-MET encouraged us to investigate the changes in their in vivo bio-absorption further. Therefore, an in vivo pharmacokinetic study in rats was conducted. The results showed that salt formation substantially increased the absorption rate of NSAIDs, which is important for developing new dosage forms of drugs, developing other pharmacological activities, and providing additional routes of administration.

## 2. Materials and Methods

### 2.1. Materials

Ketoprofen (purity > 98%), phenylbutazone (purity > 98%) and metformin–HCl (purity > 98%) were purchased from Jiuding Chemistry Biotechnology Co., Ltd. (Shanghai, China). All other reagents were of analytical grade and commercially available.

### 2.2. Salt Synthesis

**Figure 1:**
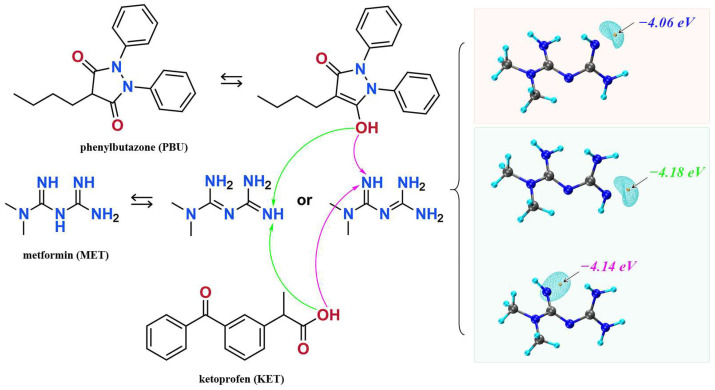
The molecular structures of ketoprofen, phenylbutazone, and metformin and possible mechanisms of proton transfer.

We synthesized NSAIDs–MET through liquid-assisted griding of the mixture of NSAIDs and MET with a 1:1 stoichiometric ratio, which was ground with 2 mL methanol for approximately 20 min. About 80 mg of the powdered sample of NSAIDs–MET was dissolved in 6 mL methanol–water. Subsequently, the solution was filtered and crystallized after five days. Fine block-shaped crystals, which were suitable for SCXRD, were obtained by slow evaporation. Figure 1 displays the molecular structures and possible mechanisms of proton transfer of the compounds.

### 2.3. Characterization

#### 2.3.1. Single Crystal X-ray Diffraction (SCXRD) Analysis

Single crystal X-ray data were measured on a Rigaku XtaLAB Synergy four-circle diffractometer using Cu Kα radiation (λ = 1.54178 Å) (Rigaku, The Woodlands, TX, USA). All intensity data were collected at 293 K. Data were corrected for absorption effects using the CrystalClear software (https://www.rigaku.com/downloads/software/crystalclear/index.html (accessed on 4 March 2024)) (Rigaku, USA). Crystal structures were solved using direct methods and refined employing the SHELXL and Olex2 suite of programs, and the final refinements were performed using the full-matrix least-squares methods [27,28,29]. All non-hydrogen atoms were refined anisotropically. Hydrogen atoms connected to carbon, nitrogen, and oxygen atoms were all placed in idealized positions.

#### 2.3.2. Powder X-ray Diffraction (PXRD) Analysis

PXRD experiments were performed on a Rigaku D/max-2550 powder X-ray diffractometer with Cu Kα radiation (λ = 1.54178 Å) (Rigaku, Tokyo, Japan). The powder samples were scanned continuously with a coverage of 3–40° at a constant rate of 8°/min. Simulated PXRD patterns were calculated using Mercury software (v2023.1.0, Cambridge Crystallo-graphic Data Center, Cambridge, UK) at a starting angle of 3°, a final angle of 40°, a step size of 0.02°, and a full width at half maximum of 0.15°.

#### 2.3.3. Differential Scanning Calorimetry (DSC) Analysis

DSC thermograms were recorded with DSC 1 (Mettler Toledo, Greifensee, Switzerland) and STARe Evaluation software 16.0. Approximately 3–5 mg was weighed into an aluminum crucible, sealed using a lid with a hole, and then heated at a constant rate of 10 °C/min over a temperature range of 30–300 °C under atmospheric conditions.

#### 2.3.4. Infrared Spectroscopy (IR) Analysis

IR experiments were performed on a Spectrum 400 Fourier transform infrared spectrometer (PerkinElmer, Waltham, MA, USA). The experimental conditions included an attenuated total reflection accessory, a spectral scanning range of 4000–400 cm^−1^, a resolution of 4.000 cm^−1^, and a scan number of 16.

### 2.4. Solubility Experiments

Samples for the solubility studies were prepared following the shake-flask method [30]. The test samples were pre-sieved through a 100 mesh sieve to obtain powders with similar particle size ranges. Saturated solutions were obtained by stirring an excess of NSAIDs and NSAIDs-MET in 1 mL of buffer at pH 1.2, pH 4.5, and pH 6.8 and water at 25 °C. After 48 h, the samples were filtered through 0.22 µm microporous membrane filters and measured directly using high-performance liquid chromatography (HPLC). The experiments were repeated three times.

The concentrations of NSAIDs were quantified on an Agilent high-performance liquid chromatography system (Agilent 1260 series, Jersey City, NJ, USA) with an Odyssil C18 column (4.6 mm × 250 mm, 3 µm). The mobile phase was prepared with acetonitrile-1% glacial acetic acid (70:30), the flow rate was 1 mL·min^−1^, and the column temperature was set at 30 °C.

### 2.5. Intrinsic Dissolution Rate (IDR) Experiments

Samples containing 150 mg of NSAIDs (in equivalence) were compacted into round discs of 8 mm diameter using a flat-faced round punch (FU KESI, Shanghai, China). The static disc method was performed at 100 rpm in 900 mL of pH 6.8 buffer at 37 ± 1 °C for 30 min. At specific time intervals, 1.5 mL of each solution was collected as a sample, and an equal volume of fresh buffer was added immediately. The obtained solutions were filtered through a 0.22 µm microporous membrane filter and measured directly using HPLC. See Section 2.4 for the experimental conditions. The experiments were repeated three times.

### 2.6. Permeability Experiments

Permeability experiments with NSAIDAs and NSAIDAs-MET were measured by the modified Franz diffusion cell apparatus through a cellulose nitrate membrane (0.45 µm, Cytiva, Freiburg im Breisgau, Germany). The membrane was placed in between the donor and recipient compartment, to which 5 mL of a buffer medium (pH 6.8) was added. After the buffer medium was kept at 37 ± 0.2 °C and rotated at 100 ± 5 rpm, approximately 15 mg of NSAIDs (in equivalence) were placed on the membrane. At predetermined time intervals, 0.5 mL of the sample was withdrawn from the receptor compartment and replaced with fresh medium. Finally, the concentration of NSAIDs was analyzed by HPLC. See Section 2.4 for experimental conditions. The experiments were repeated three times.

The apparent permeability coefficient (P_app_) of oral drug-permeable membranes can represent the magnitude of drug transport capacity, and the formula is shown below [31].

P_app_ = (dQ/dt)/(A × C_0_) (1)

where P_app_ is in cm·s^−1^, dQ/dt is the drug transport per unit time (μg·min^−1^), A is the surface area of the membrane, and C_0_ is the initial concentration (μg·min^−1^). The cumulative drug transport concentration, TR_cum_, was corrected for the fact that rehydration after each sample diluted the drug permeation.

(2) $${\mathrm{TR}}_{\mathrm{cum}}=A_{n}+V_{\mathrm{Sn}}/V_{R}\;\times \;\sum_{i=0}^{n-1}A_{i}$$

where A_n_ is the measured permeability value for the nth sample, V_Sn_ is the sampling volume, and V_R_ is the receiving cell volume.

### 2.7. Dynamic Vapor Sorption (DVS) Experiment

The hygroscopicity of NSAIDs–MET was studied based on a dynamic vapor sorption experiment (DVS Adventure, Surface Measurement Systems, London, UK). The samples were studied at 25 °C in the humidity range of 0–90% relative humidity (RH). Each humidity step was performed when a change in weight of less than 0.02% occurred within 10 min, with a maximum retention time of 120 min.

### 2.8. Theoretical Calculation

Theoretical computations were conducted using density functional theory with the Gaussian 16 program [32]. Geometric optimization was performed exclusively on the hydrogen atoms at the B3LYP-D3/6-311G (d, p) level, while the heavy atoms were held at their original X-ray coordinates. Single-point energies were calculated at the B3LYP-D3/6-311+G (2d, 2p) level [33]. Wavefunction analysis was carried out using the Multiwfn 3.8 software [34]. The voids of the crystals were calculated using a mercury void module, a probe radius = 1.2 Å, and a grid spacing = 0.3 Å [35].

### 2.9. In Vivo Pharmacokinetic Study

A total of 20 male Sprague Dawley rats (230 ± 20 g) were supplied by the Experimental Animal Center of the Institute of Materia Medica, Chinese Academy of Medical Sciences. Animals were housed and handled under suitable humidity, temperature, and light. The rats were allowed to acclimate for one week with free access to water and standard rodent food. This study was approved by the Animal Ethics Committee of the institution and conducted in accordance with the Guideline for Animal Experimentation of the Institute of Materia Medica, Chinese Academy of Medical Sciences.

The rats were randomly divided into four groups (*n* = 5 per group), and NSAIDs (100 mg/kg) or NSAIDs-MET were administered to each rat. After administration, 400 μL blood samples were collected through the retro-orbital venous plexus at 0, 5, 15, 30, 60, 120, 180, 240, 360, 480, 600, 720, and 1440 min. The samples were centrifuged at 5000 rpm (10 min), and the supernatants of samples were stored at −80 °C until analysis.

After the plasma samples were thawed at room temperature, 100 μL of plasma was mixed with 20 μL of aminopyralid or naproxen solution (100 μg/mL, as the internal standard, IS) in a 1.5 mL EP tube. After 1 min of mixing and vortexing, 1 mL of ethyl acetate was added. The mixture was centrifuged at 5000 rpm for 10 min, and 800 μL of supernatant was separated and blown dry under nitrogen at 40 °C. The supernatant was then dried under nitrogen at 40 °C. After adding 50 μL of methanol, vortexing for 1 min, and centrifuging for 10 min at 12,000 rpm, 20 μL of the supernatant was extracted and analyzed by HPLC. Plasma concentration–time curves and some important pharmacokinetic parameters were obtained using DAS 2.0 software. The data obtained were expressed as mean ± standard deviation (mean ± SD).

## 3. Results and Discussions

### 3.1. Characterization Analysis

#### 3.1.1. SCXRD Analysis

**Figure 2:**
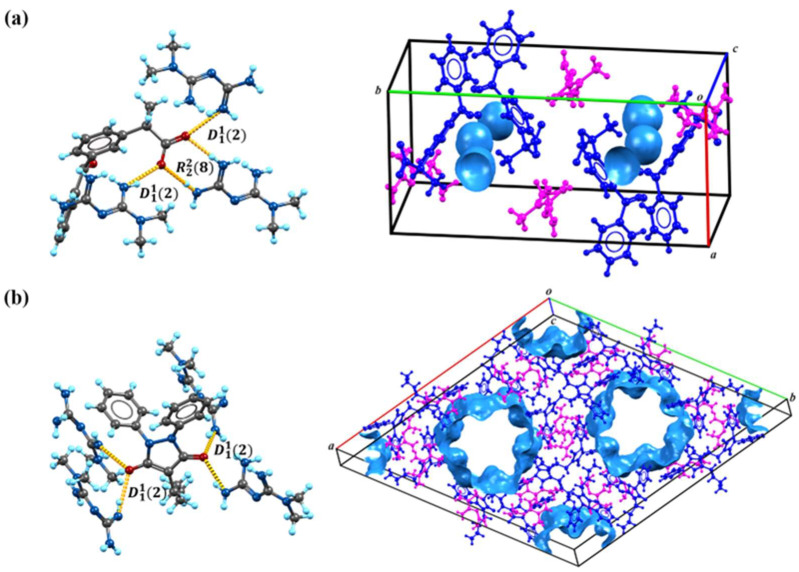
The H bond motifs between API and MET (left), packing, and voids of the crystal structures (right). (**a**) KET-MET; (**b**) PBU-MET.

Based on the calculations in Figure 1, we speculate on the potential sites of proton transfer. The pyrazolidinedione in the structure of PBU can resonate from the keto reciprocal to the enol form, which undergoes proton transfer [36]. The proton transfer of KET occurs at the hydrogen on the carboxyl group. The MET configuration has three possible sites for proton acquisition: the orange blob indicates the global minima of the electrostatic potential, and the blue grid iso-surface shows the iso-surface map of the electrostatic potential. Calculations show that the two sites of the structure in the green box have closer minima, and it is hypothesized that protons should tend to transfer to this site and form hydrogen bonds with the corresponding compounds, as confirmed in single crystals. Table 1 lists the detailed crystallographic information for the salts. In this, the O_1_ atom of KET-MET, as well as the C_6_ and C_7_ atoms of PBU-MET, were made to be disorderly. Figure 2 shows the H bond motifs between API and MET, packing, and voids of the crystal structures, where the blue part on the right indicates the solvent accessible volume. The KET-MET void volume is 2.2% of then unit cell volume, while PBU-MET is 29.7% of the unit cell volume.

KET-MET crystallizes in a 1:1 stoichiometric ratio in a monoclinic crystal system with space group ${P2}_{1}/c\;$(Z = 4). Each asymmetric unit contains one KET cation and one MET anion. Consistent with speculative results, proton transfer occurs from the carboxyl acid group of KET to the N_4_ atom of the MET molecule, thus resulting in the formation of a charge-assisted N_4_-H_4A_···O_2_ ionic interaction (Figure 2a). KET is hydrogen-bonded to neighboring METs via N_1_-H_1A_···O_3_ and N_1_-H_1B_···O_3_ to form the $D_{1}^{1}$(2) motif. The two asymmetric units of KET-MET form a centrosymmetric dimer via hydrogen bonding to N_1_-H_1A_···O_3_ and N_2_-H_2A_···O_2_ ($R_{2}^{2}$(8) motif). The dimer is connected by N_2_-H_2B_···N_3_ hydrogen bonds between neighboring METs to form chains extending along the c-axis. These chains are connected by N_4_-H_4A_···O_2_ hydrogen bonds between the MET and the KET to form a planar structure. The stacking of these hydrogen bonding interactions forms the three-dimensional structure.

PBU-MET crystallizes in a 1:1 stoichiometric ratio in a trigonal with the space group $R\bar{3}\;$(Z = 18). Upon binding to metformin, each asymmetric unit consists of a PBU cation and a MET anion. Protons are transferred from O_2_ on the enolitic structure of the PBU to the N_4_ atoms of the MET molecule, thus resulting in the formation of a charge-assisted N_4_-H_4D_···O_2_ ionic interaction (Figure 2b). The two asymmetric units of PBU-MET form a chain structure along the a-axis through N_3_-H_3B_···O_1_, N_4_-H_4C_···O_2_, and N_4_-H_4D_···O_2_ hydrogen bonds. These chains are interconnected through N_6_-H_6C_···O_1_ hydrogen bonds between PBU and MET ($D_{1}^{1}$(2) motif) to form a planar structure. According to the calculations, the void volume of PBU-MET accounts for about one-third of the unit cell volume. The molecule forms a supramolecular structure with a special void structure by stacking, and the unique void structure provides the basis for its enhanced solubility.

#### 3.1.2. PXRD Analysis

**Figure 3:**
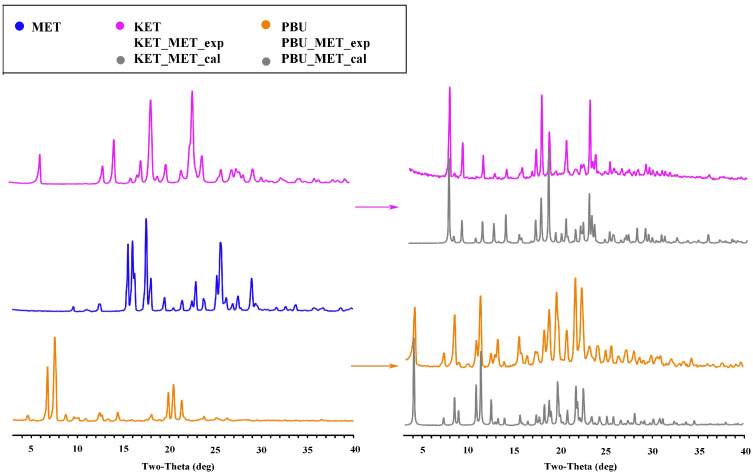
PXRD patterns for MET, NSAIDs, and the corresponding salts. Experimental patterns of metformin and NSAIDs, experimental patterns of NSAIDs-MET, and calculation patterns of NSAIDs-MET.

As a mature approach, PXRD is commonly used in the structural characterization of polymorphic substances, which can give important information on the formation, purity, and crystallization degree [37]. Figure 3 shows the PXRD patterns of the NSAID-MET samples obtained using the liquid-assisted grinding method. The PXRD patterns showed significant differences in the number, intensity, and topological profile of the diffraction peaks, and these differences proved the formation of a new phase. Additionally, Figure 3 depicts a high degree of fit of the sample PXRD profile to the simulated profile of the SCXRD data. The results indicate the crystallinity and high purity of the prepared samples and can be used for subsequent experimental studies.

#### 3.1.3. DSC Analysis

**Figure 4:**
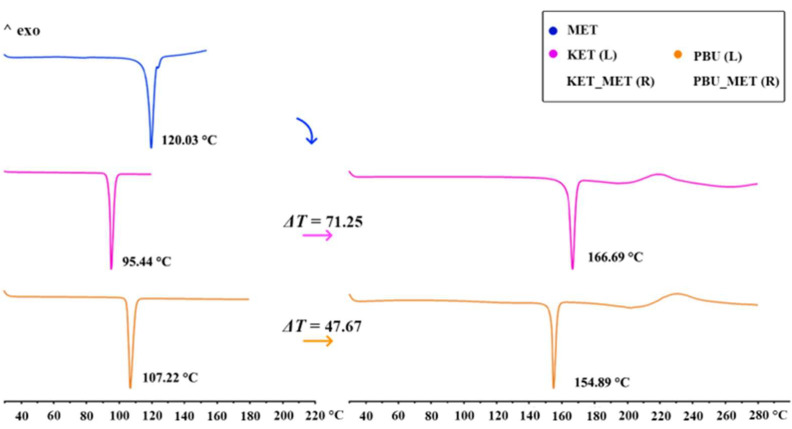
DSC patterns for MET, NSAIDs (**Left**), and the corresponding salts (**Right**).

DSC was used to assess the thermal stability of the new phase and to determine its melting point. The figure shows the thermal characteristics of NSAIDs-MET obtained through DSC. As can be seen from Figure 4, the KET-MET and PBU-MET salts exhibited only a single heat absorption peak at 166.69 °C and 154.89 °C, indicating the absence of solvent molecules, which is consistent with the SCXRD results. Furthermore, in contrast to MET and NSAIDs, all salts exhibited higher melting points, indicating higher thermodynamic stability [38]. This may be due to the formation of charge-assisted hydrogen bonds, which are stronger with high interaction strengths and exhibit an increased melting point. Theoretical calculations of the electron densities at BCP (+3, −1) and the estimated strength for the major hydrogen bond proved our conjecture [39]. The melting point was positively correlated with the hydrogen bonding strength and interaction energy, with stronger hydrogen bonding strength and interaction energy showing higher melting points. Consistent with the results of the DSC experiments, a significant increase was observed in the hydrogen bond strength and interaction energy after the formation of the salt, which is manifested by the increase in the melting point of NSAIDs-MET. The hydrogen bond strengths (N-H···O, −13.22 kJ/mol) and interaction energies of KET-MET (−93.83 kJ/mol) are higher than those of PBU-MET (−10.36 and −84.49 kJ/mol), which is consistent with the fact that the melting point of KET-MET is higher than that of PBU-MET, indicating that KET-MET is more thermally stable.

#### 3.1.4. IR Analysis

**Figure 5:**
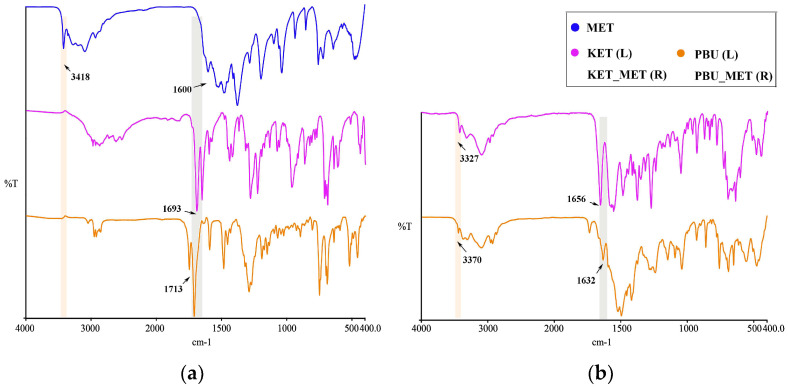
IR characteristics for NSAIDs and NSAIDs-MET. (**a**) MET and NSAIDs; (**b**) NSAIDs-MET.

Infrared spectroscopy is a fundamental tool for functional group identification of molecular assemblies based on the physical state and hydrogen bonding interactions [40]. Changes in these groups, such as the formation of a new solid state resulting in hydrogen bonding, influence the vibrational modes associated with the functional groups. Consequently, IR studies of NSAIDs and NSAIDs-MET were performed as shown in Figure 5. At approximately 1715 cm^–1^, pure NSAIDs exhibit a distinct peak associated with the C=O stretching vibration. NSAIDs-MET salts absorb weakly in the range of 1680–1720 cm^−1^ and strongly at 1600–1660 cm^−1^. The asymmetric C-O stretching of the carboxylic acid was observed to move towards lower wavelengths, indicating that the carboxylic acid is converted to carboxylate ions through interactions with NSAIDs and MET. Thus, this characteristic spectrum could suggest the formation of carboxylate salts. Furthermore, this is similarly supported by the appearance of a broad band of carboxylates at approximately 3100–2500 cm^−1^ in the spectra of NSAIDs-MET.

Similarly, metformin N-H stretching vibration occurred at 3418 cm^−1^. After the formation of NSAIDs-MET, N-H stretching vibration appeared at 3327 and 3370 cm^−1^, respectively. There is a shift compared to a single NH_2_ wave number. The guanidine group exhibited a C=N stretching pattern between 1580 and 1685 cm^−1^ [41], which occurred at 1600 cm^−1^ in the experiment, which is very close to the reported value. In contrast to the C=N stretching vibration of metformin, the C=N stretching pattern in the NSAID-MET correlated state moved towards a lower wave number, demonstrating the newly formed intermolecular hydrogen bond between the two molecules.

### 3.2. Solubility Studies

**Figure 6:**
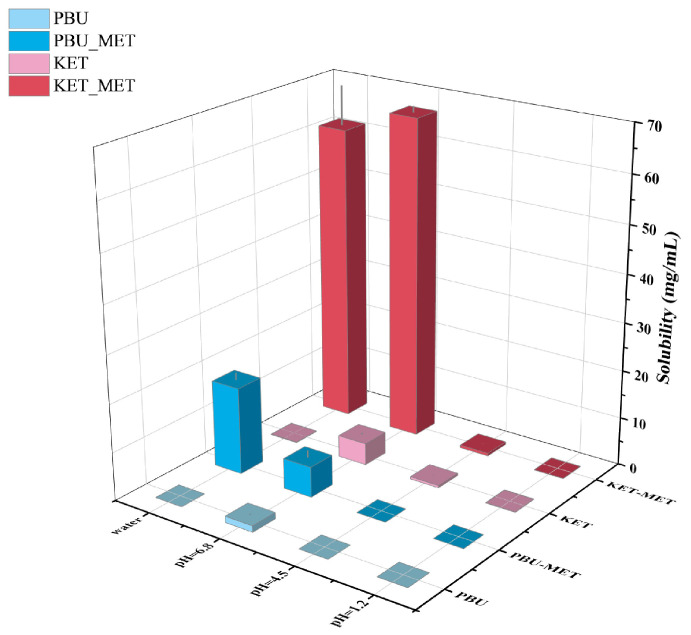
Solubility statistics of NSAIDs and NSAIDs-MET at different pH conditions.

As one of the factors affecting drug absorption, transport, metabolism, and excretion, solubility is a fundamental and major challenge for researchers attempting to manipulate and evaluate drug properties throughout the drug design and development process [42]. Solubility is closely related to oral bioavailability; thus, increasing solubility is a common way to increase the bioavailability of insoluble drugs. Herein, the solubility of NSAIDs and NSAIDs-MET was investigated by suspending excess solids in different buffers. Figure 6 and Table 2 indicate that the solubility of NSAIDs and NSAIDs-MET increases as the pH of the buffer increases. NSAIDs have the highest solubility in buffer media at pH 6.8, while their solubility in water is low, corresponding to poor water solubility. Although NSAIDs-MET did not increase the solubility of NSAIDs in buffers at pH 1.2 and 4.5, it was considerably soluble at pH 6.8 and in water. The solubility of KET-MET in pH 6.8 buffer and water is 14.7 times and 522.6 times that of KET. Compared with PBU, the solubility of PBU-MET in water is increased by an astonishing 3630 times, significantly improving the water solubility of insoluble NSAIDs. This may be due to the formation of a salt; API and SF are in the ionic state and are more likely to dissociate by interacting with solvents such as water, thus providing the basis for increased solubility. This also explains the low solubility of NSAIDs-MET under acidic conditions, mainly because the NSAIDs ions in it revert to a neutral molecular state. Thus, the solubility is essentially the same as that of the raw material [43]. Moreover, the molecules are looser due to the void structure in the molecular structure of PBU, which is another reason for its high solubility. It is worth noting that pH 6.8 represents the pH environment present in human small intestines, where most absorption occurs. Accordingly, the increased solubility at pH 6.8 may provide the basis for more adequate absorption [44]. Furthermore, the satisfactory solubility characteristics and solution stability of these salts serve as the basis for subsequent IDR and permeability studies.

### 3.3. Intrinsic Dissolution Rate (IDR) Studies

**Figure 7:**
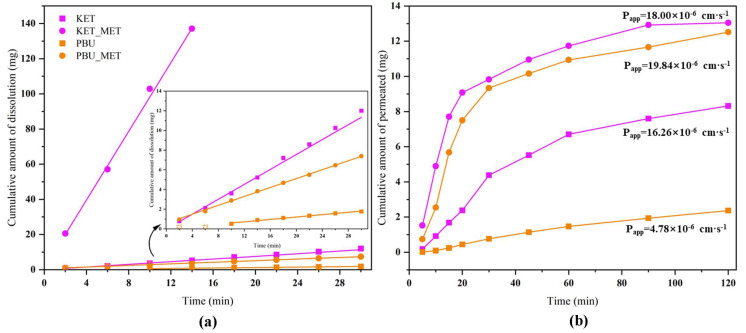
Cumulative amount of dissolution (**a**) and cumulative amount of permeated (**b**) NSAIDs-MET and NSAIDs vs. time plot.

We conducted IDR studies from the dynamics perspective to ascertain the degree to which NSAID-MET salts influence the dissolution rate of pure NSAIDs. This research established a scientific basis for modifying the in vivo properties of the salt. The results provided by solubility showed a great enhancement in the pH 6.8 buffer; therefore, the IDR experiment was conducted in this condition. Since KET-MET dissolves completely in approximately 15 min, only the first 15 min of sampling time points were considered in the calculation. PBU was not detected at concentrations in the first 6 min due to poor solubility and was also not considered in the calculations. Figure 7a displays the calculated IDR average values of NSAIDs-MET at pH 6.8 compared to pure NSAIDs. The results showed that both the dissolution rate and the cumulative dissolution amount of NSAIDs-MET were more increased than those of NSAIDs alone. The dissolution rate of KET-MET was astonishingly 25.8 times higher than that of KET. The reason for the substantial increase in the dissolution rate of NSAIDs-MET may be that when the salt is immersed in an aqueous environment, due to the good aqueous solubility of MET, it can be dissolved into solution first, resulting in the disintegration of the NSAID-MET salt. The amorphous-like state of the NSAID molecules, which has a much higher internal energy, is exposed to a solvent and may be rapidly dispersed and dissolved in solution [45]. Overall, the results of the IDR experiments were consistent with those of the solubility experiments. NSAIDs-MET had a higher IDR and showed greater advantages than NSAIDs in solubility, suggesting that NSAIDs-MET has great potential for improving the pharmacokinetics and bioavailability in vivo.

### 3.4. Permeability Studies

Maintaining the balance between solubility and permeability is a key task for drugs [46]. Membrane permeability is a major determinant in the drug transport, especially during absorption from the administration site to the blood and distribution from the blood to tissues [47]. Consequently, the satisfactory dissolution behavior encouraged us to investigate the changes in permeability of NSAID-MET salts further to evaluate the effect of elevated dissolution characteristics on permeability. To this end, permeability experiments with NSAID-MET salts were performed using a modified Franz diffusion device [26]. Figure 7b illustrates the plot with the average of the results. The highest cumulative drug diffusion rate (1.593 mg·cm^−1^·min^−1^) was observed for PBU-MET at 30 min, which was 11.3 times higher than that of PBU. In addition, the apparent permeability coefficient of PBU-MET is 4.15 times that of PBU. Although KET itself has excellent permeability, its P_app_ has increased after the formation of KET-MET, which is 1.1 times greater than that of KET. It proved that the membrane diffusion ability of NSAIDs was enhanced after salt formation with MET. Interestingly, consistent with the results of solubility and IDR experiments, the amount of salt diffusing through the membrane was KET-MET > PBU-MET. The increased permeability of NSAIDs in salt may be attributed to the improved solubility of MET, which may lead to higher concentration gradients across the membrane as the driving force. In addition, there are two possible reasons for the change in permeability based on changes in molecular structure. The first reason is the possible change in lipophilicity after salt formation [48]. The higher the log *p* value, the relatively higher the concentration in the lipid phase and the higher the lipid solubility, and therefore, the salt exhibits a higher permeability. The other reason is that the introduction of metformin makes the spatial site resistance larger, causing an increase in permeability, or the electronegative group of metformin enhances its permeability [49]. Although NSAIDs themselves belong to BCS class II, which has better permeability, the increased permeability in this study may provide more options for the route of administration and the possibility of developing different active pharmaceuticals [50]. Since NSAIDs-MET provides an excellent basis for enhancing solubility and permeability, this encourages further in vivo bioavailability evaluation.

### 3.5. DVS Studies

**Figure 8:**
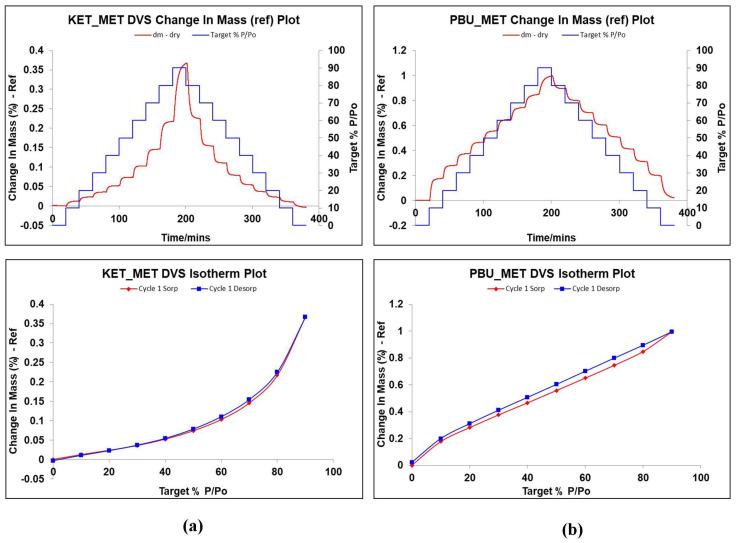
Changes in hygroscopic mass gain and adsorption desorption of NSAIDs at 25 °C with relative humidity of 0–90%. (**a**) KET-MET; (**b**) PBU-MET.

DVS is a dynamic analysis used to study the changes in drug weight caused by water absorption as humidity increases. It is known from the literature that MET is highly hygroscopic. It absorbs a large amount of water after 60% RH, and the weight change is approximately 80%, leading to the deliquescence of the drug in a short period due to water adsorption [51,52,53]. Given the extremely strong hygroscopicity of MET, the investigation and validation of the hygroscopicity of NSAIDs-MET after salt formation was an important part of our study. Figure 8 displays the weight change values of NSAIDs-MET in the range of 0–90% relative humidity, which can be observed to determine the hygroscopic behavior in air. Surprisingly, when MET and NSAIDs were combined to form a multi-component salt, the weight of the sample did not change by more than 1% even at 90% relative humidity. This indicates that the introduction of metformin does not introduce its own hygroscopic properties into the new product while improving its physicochemical properties, such as solubility, permeability, and stability, and the new compound can be stably stored at room temperature and relative humidity.

### 3.6. In Vivo Pharmacokinetics

**Figure 9:**
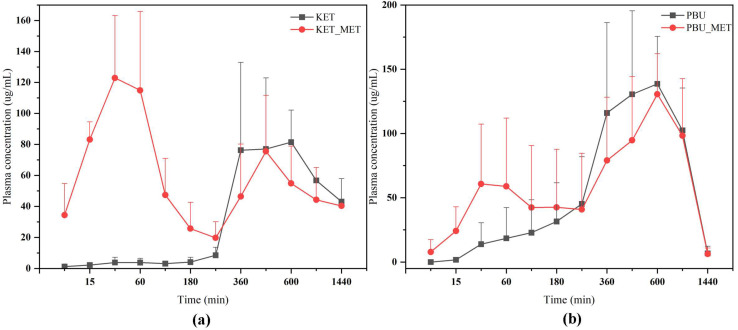
Plasma concentration–time profiles of NSAIDs and NSAIDs-MET after oral administration. (**a**) KET-MET; (**b**) PBU-MET.

The plasma concentration–time profiles of NSAIDs and NSAIDs-MET after oral administration are shown in Figure 9. The calculated pharmacokinetic parameters are summarized in Table 3. The C_max_ of KET-MET (125.77 ± 39.89 mg/L) was elevated compared to the pure KET (112.00 ± 17.05 mg/L), whereas the C_max_ of PBU-MET was slightly decreased in comparison to the PBU. The bioavailability of KET in KET-MET was 114% of KET alone, as measured by AUC_0-∞_. Contrary to the predictions made by prior experimental findings, the in vivo bioavailability of PBU was diminished although PBU-MET enhanced its solubility and permeability in vitro. T_1/2_ delays of the NSAIDs in NSAIDs-MET suggest that salt formation can affect the duration of action of NSAIDs in vivo. Despite the decreased bioavailability of PBU-MET, the increase in MRT_0-∞_ and decrease in CL_z_/F suggest that PBU-MET has a longer retention time and slower clearance in vivo than pure PBU, which facilitates the efficacy of PBU in vivo and has a higher safety profile. Interestingly, the plasma concentration–time profiles of KET-MET and PBU-MET showed unique bio-absorption properties. This may be because after solid gavage administration, some of the salts are destroyed in the acidic environment of the stomach, and the undestroyed salts are rapidly absorbed through the intestines, showing higher blood concentrations in the pre-drug period. The drug with a destroyed salt structure showed slower bio-absorption, consistent with the shape of the plasma concentration–time graph of NSAID APIs. Furthermore, the in vitro solubility evaluation experiments proved our speculations. NSAIDs-MET showed poor solubility in the simulated gastric acid pH environment, whereas in the simulated intestinal pH environment, NSAIDs-MET showed a significant improvement in solubility than NSAIDs. Therefore, this implies that the preparation of salt enteric-soluble formulations, which maintain the drug in salt form to facilitate intestinal absorption, might be crucial in enhancing drug bioavailability and showing a rapid anti-inflammatory response. This is essential for future research.

## 4. Conclusions

The structures of NSAIDs and NSAIDs-MET were characterized using SCXRD, PXRD, DSC, and IR. The melting points of the drug multicomponent salts were significantly higher than those of the two APIs, indicating higher thermal stability after salt formation. The results of the DVS study demonstrated that the introduction of metformin improved the physicochemical properties of NSAIDs-MET without inducing a high hygroscopicity similar to that of metformin. The solubility results showed that NSAIDs-MET could significantly enhance the solubility and intrinsic dissolution rate of NSAIDs in a pH 6.8 medium and water. The permeability experiments based on increasing the solubility of NSAIDs-MET showed that the P_app_ of the KET-MET and PBU-MET salts were 1.1 and 4.15 times higher than those of the salts, effectively improving permeability. The simultaneous enhancement of solubility and permeability may be associated with safety issues. However, it simultaneously provides more basis for lowering the drug dosage or developing other active formulations. This is an in vitro method that mimics the in vivo environment, and its in vitro–in vivo correlation needs further validation. Perhaps performing cell permeability evaluation experiments such as Caco-2 and MDCK would be a more desirable approach. The in vivo pharmacokinetic experiments showed that maintaining the salt state significantly increased the absorption rate of NSAIDs, allowing them to show rapid anti-inflammatory effects and high bioavailability. Considering that the salt may be destroyed in the stomach, the preparation of enteric formulations may overcome this obstacle, and this will also be an important direction for our future research. We demonstrated that NSAIDs-MET significantly improved the thermal stability, solubility, permeability, and bio-absorption rate of NSAIDs. This provided a research basis for developing new drug multicomponent solids and designing new dosage forms.

## Figures and Tables

**Table 1:** Crystal cell parameters and structure refinement of the salts.

	KET-MET	PBU-MET
Formula	C_16_H_13_O_3_·C_4_H_12_N_5_	C_19_H_19_N_2_O_2_·C_4_H_12_N_5_
Crystal size (mm)	0.20 × 0.20 × 0.20	0.20 × 0.20 × 0.20
Molecular weight	383.45	436.74
Temperature (K)	293 (2)	293 (2)
Crystal system	monoclinic	trigonal
Space group	$P2_{1}/c$	$R\bar{3}$
α (Å)	10.513 (1)	40.224 (1)
b (Å)	22.394 (2)	40.224 (1)
c (Å)	8.807 (1)	9.958 (1)
a (deg)	90	90
β (deg)	93.628 (5)	90
γ (deg)	90	120
Volume (Å3)	2069.45 (19)	13,953.5 (7)
Z	4	18
Density (g/cm^3^)	1.231	0.936
R1 (I 2σ(I))	0.0656	0.0624
wR2 (I 3σ(I))	0.1798	0.1770
Goodness-of-fit on F2	1.039	1.058
Completeness (%)	99.9%	98.9%
CCDC deposition no.	2,324,339	2,324,340

**Table 2:** Solubility of NSAIDs and NSAIDs-MET in pH 1.2, pH 4.5, and pH 6.8 buffer and water.

Solubility (mg/mL)				
Compound	pH 1.2 Buffer	pH 4.5 Buffer	pH 6.8 Buffer	Water
KET KET-MET	0.076 ± 0.0358	0.494 ± 0.0394	4.555 ± 0.1932	0.118 ± 0.0529
	0.049 ± 0.0433	0.567 ± 0.0278	66.987 ± 1.2080	61.668 ± 8.0857
PBU PBU-MET	0.006 ± 0.0022	0.001 ± 0.0006	1.326 ± 0.0849	0.005 ± 0.0041
	0.007 ± 0.0007	0.010 ± 0.0075	6.327 ± 1.3960	18.152 ± 1.4396

**Table 3:** Pharmacokinetic parameters after oral administration of NSAIDs and NSAIDs-MET in rats (x ± s, *n* = 5).

Parameter	KET	KET-MET	PBU	PBU-MET
AUC_0-t_ (mg/L*h)	1150.30 ± 133.78	1214.72 ± 305.84	1593.36 ± 457.03	1532.80 ± 501.89
AUC_0-∞_ (mg/L*h)	1995.02 ± 1011.62	2273.14 ± 155.47	1631.16 ± 435.64	1570.51 ± 504.48
MRT_0-t_ (h)	12.87 ± 0.90	11.27 ± 1.22	9.75 ± 1.68	9.78 ± 1.74
MRT_0-∞_ (h)	26.16 ± 10.27	25.01 ± 2.14	10.25 ± 2.07	10.30 ± 2.41
t_1/2z_ (h)	13.04 ± 8.75	15.90 ± 1.65	3.07 ± 120	3.59 ± 1.38
T_max_ (h)	8.00 ± 2.00	0.67 ± 0.29	8.00 ± 2.00	9.60 ± 1.67
CL_z_/F (L/h/kg)	0.06 ± 0.02	0.07 ± 0.01	0.10 ± 0.04	0.07 ± 0.02
V_z_/F (L/kg)	0.90 ± 0.34	1.519 ± 0.05	0.30 ± 0.20	0.49 ± 0.15
C_max_ (mg/L)	112.00 ± 17.05	125.77 ± 39.89	164.76 ± 43.81	149.04 ± 28.52

## Data Availability

All experimental data required to reproduce the findings from this study will be made available to interested investigators.
